# SUBJECTIVE SOCIAL STATUS MODERATES BACK PAIN AND MENTAL HEALTH: A LONGITUDINAL ANALYSIS OF OLDER MEN

**DOI:** 10.1093/geroni/igac059.1933

**Published:** 2022-12-20

**Authors:** Christina Mu, Dylan Jester, Peggy Cawthon, Katie Stone, Nancy Lane, Soomi Lee

**Affiliations:** University of South Florida, Tampa, Florida, United States; University of California San Diego, La Jolla, California, United States; California Pacific Medical Center Research Institute, San Francisco, California, United States; University of California, San Francisco, Castro Valley, California, United States; University of California, Davis Health, Davis, California, United States; University of South Florida, Tampa, Florida, United States

## Abstract

Objectives. This study tested the longitudinal relationship between back pain and mental health and examined the moderating role of subjective social status (SSS). Method. Community-dwelling older men from the MrOS Study provided four study visits of data collected between 2000-2016 (15,975 observations nested within 5,979 participants). Back pain frequency and severity were assessed at visits 1-4. General mental health was measured at each visit by the 12-item Short Form Survey Mental Component Score (SF-12 MCS; higher scores representing better mental health). National and community SSS were assessed at visits 1 and 3 with the MacArthur Scale. Growth curve models tested longitudinal within-person change associations after accounting for the repeated measures within each person. Age was used as the primary time variable. Results. At baseline, those with higher back pain-frequency/severity reported lower SF-12 MCS. After accounting for this between-person difference, there were bidirectional within-person associations between back pain frequency/severity and SF-12 MCS. On follow-up visits when back pain frequency/severity increased from baseline, participants reported lower SF-12 MCS (p<.001). On follow-up visits when SF-12 MCS decreased from baseline, participants also reported higher back pain frequency/severity (p<.001). Higher national and community SSS at baseline and having increases or consistently higher SSS over time attenuated the negative relationships between back pain frequency/severity and SF-12 MCS. Results were consistent after controlling for an extensive list of baseline health covariates and pain medications. Discussion. These findings highlight how self-perceived social status may buffer the relationship between greater back pain frequency/severity and lower mental health.

